# CXCL2 Impairs Functions of Bone Marrow Mesenchymal Stem Cells and Can Serve as a Serum Marker in High-Fat Diet-Fed Rats

**DOI:** 10.3389/fcell.2021.687942

**Published:** 2021-07-13

**Authors:** Jianhai Bi, Qiuchen Li, Zhigang Yang, Lei Cai, Tao Lv, Xun Yang, Li Yan, Xia Liu, Qian Wang, Xin Fu, Ran Xiao

**Affiliations:** ^1^Research Center of Plastic Surgery Hospital, Chinese Academy of Medical Sciences and Peking Union Medical College, Beijing, China; ^2^Shandong Provincial Hospital Affiliated to Shandong First Medical University, Jinan, China

**Keywords:** bone marrow mesenchymal stem cells, cytokine, CXCL2, high-fat diet, inflammation

## Abstract

In modern society excessive consumption of a high-fat diet (HFD) is a significant risk factor for many diseases such as diabetes, osteoarthritis and certain cancers. Resolving cellular and molecular mechanisms underlying HFD-associated disorders is of great importance to human health. Mesenchymal stem cells (MSCs) are key players in tissue homeostasis and adversely affected by prolonged HFD feeding. Low-grade systemic inflammation induced by HFD is characterized by increased levels of pro-inflammatory cytokines and alters homeostasis in many organs. However, whether, which and how HFD associated inflammatory cytokines impair MSCs remain unclear. Here we demonstrated that HFD induced serum cytokines disturbances, especially a continuous elevation of serum CXCL2 level in rats. Coincidentally, the differentially expressed genes (DEGs) of bone marrow MSCs (BMSCs) which functions were impaired in HFD rats were enriched in cytokine signaling. Further mechanism analysis revealed that CXCL2 treatment in vitro suppresses the adipogenic potential of BMSCs via Rac1 activation, and promoted BMSC migration and senescence by inducing over-production of ELMO1 and reactive oxygen species (ROS) respectively. Moreover, we found that although glycolipid metabolism indicators can be corrected, the CXCL2 elevation and BMSC dysfunctions cannot be fully rescued by diet correction and anti-inflammatory aspirin treatment, indicating the long-lasting deleterious effects of HFD on serum CXCL2 levels and BMSC functions. Altogether, our findings identify CXCL2 as an important regulator in BMSCs functions and may serve as a serum marker to indicate the BMSC dysfunctions induced by HFD. In addition, our findings underscore the intricate link among high-fat intake, chronic inflammation and BMSC dysfunction which may facilitate development of protective strategies for HFD associated diseases.

## Introduction

In recent years, overnutrition like excessive consumption of a high-fat diet (HFD) has been viewed as a significant risk factor for the obesity and obesity-associated metabolic disorders such as insulin resistance, type 2 diabetes, fatty liver disease, osteoarthritis and cancers (Stemmer et al., 2012; Kyrou et al., 2018). And HFD-induced chronic inflammation has been deeply tied to the pathogenesis of HFD-driven metabolic diseases (Kalupahana et al., 2011; Duan et al., 2018), the increased levels of circulating pro-inflammatory cytokines [i.e., interleukin (IL)-1β, IL-6, and tumor necrosis factor (TNF)-α] resulting in low-grade systemic inflammation and metabolic disorders (Duan et al., 2018). Furthermore, certain cytokine may account for alterations in body composition in chronic diseases associated with HFD. For instance, overproduction of TNF-α plays a critical role in the development of HFD-related bone loss (Zhang K. et al., 2015), osteoarthrosis (Hamada et al., 2016) and skeletal muscle wasting (Sishi et al., 2011). Increased expression of IL-1β was reported to be strongly associated with HFD-associated brain damage (Almeida-Suhett et al., 2017). Effects of TNF-α and IL-6 on the development of kidney dysfunction was further demonstrated in HFD-fed Apo E^–/–^ mice (Tomiyama-Hanayama et al., 2009). These studies reveal the role for cytokines in tissue homeostasis and function. However, it remains unclear how the HFD-driven disturbance of cytokines impacts on tissue composition and function.

Effective functions of many adult tissues and organs depend upon innate regeneration and homeostasis which are determined by mesenchymal stem cells (MSCs) (Stappenbeck and Miyoshi, 2009; Klimczak and Kozlowska, 2016). Altered functions of MSCs have been reported in animal models and human subjects with HFD-induced obesity (Wu et al., 2013; Perez et al., 2015; Pachon-Pena et al., 2016; Ambrosi et al., 2017; Louwen et al., 2018), implying that loss of effective functional MSCs pools contributes to the impaired tissue repair potential associated with HFD (Mansilla et al., 2011). Although the mechanisms of HFD-induced MSCs dysfunction are not fully understood, inflammatory cytokines have been reported to play vital roles (Badimon and Cubedo, 2017). Growing evidence showed that pro-inflammatory cytokines can directly impair the differentiation potential of adipose-derived MSCs (ADSCs) and alter their regenerative capability in obese individuals (Louwen et al., 2018). Morrison’s group revealed that leptin acted through leptin receptor signaling to promote adipogenesis and inhibit osteogenesis by mouse bone marrow-derived MSCs (BMSCs) in response to HFD (Yue et al., 2016). However, whether, which and how HFD-regulated inflammation cytokines impair the MSCs remain incompletely understood. Hence, exploring the correlation between the HFD-regulated cytokine spectrum and the function of MSCs may help to identify a more intuitive way to evaluate the regenerative potency of certain organs, which will broaden our understanding of the detrimental effects of the HFD lifestyle.

Here, we showed that metabolic disorders were induced in HFD-fed rats, coupled with perturbed circulating cytokines levels and impaired BMSC functions. We found that the HFD-induced continuous elevation of serum C-X-C motif chemokine ligand 2 (CXCL2) correlates with the impaired functions of BMSCs, and this effect cannot be fully reversed by dietary intervention and anti-inflammatory aspirin (ASA) treatment. From a mechanistic perspective, we demonstrate *in vitro* that the elevated CXCL2 decreased the adipogenic potential of BMSCs through activating Rac1 signaling and promoted migration and senescence of BMSCs by enhancing production of ELMO1 and reactive oxidative species (ROS) respectively. Altogether, our work provides insight into the correlations between HFD-induced cytokine disturbance and declined BMSC properties, which may facilitate understanding the pathological mechanisms of diseases associated with HFD.

## Materials and Methods

### Animal Experiments

Six-week old female Sprague-Dawley rats purchased from Charles River were randomized housed in individual cages, in a 22°C temperature-controlled room and assigned into the different treatment groups. The rats in the control group and the high-fat diet (HFD) groups were fed with a normal diet (ND, GB14924.3-2001, 100% basal diet) and a customized high-fat diet (consisted of 60% basal diet, 10% sucrose, 20% lard and 10% egg yolk powder) respectively. Both ND and HFD were purchased from Beijing Keao Xieli Feed limited Company. The detailed nutrient composition of normal diet was provided in Supplementary Table 1. The rats in the dietary intervention group were fed with the HFD for 2 months followed by ND feeding for up to 6 months. A schematic figure was demonstrated in Supplementary Figure 1A. All the animal experiments were approved by the Institutional Animal Care and Use Committee of Plastic Surgery Hospital (Institute). Body weight and body length of rats (*n* = 10/group) were measured monthly for 6 months, and the Lee obesity index was calculated as $\text{LOI}=\frac{\sqrt[{3}]{\mathrm{body}\,\mathrm{weight}\,\left(\text{kg}\right)\times 1000}}{\mathrm{body}\,\mathrm{length}\,\left(\text{cm}\right)}$. All efforts were made to minimize animal suffering and to reduce the number of animals used.

### Biochemical Assessment

The rats were fasted overnight and blood glucose and cholesterol levels of rats in both groups (*n* = 5 per group) were measured using an Accu-chek active glucose meter (Roche, Basel, Switzerland) and Cobus 6000 C501 automatic biochemistry analyzer (Roche), respectively. Blood insulin levels (*n* = 5 per group) were evaluated using a rat insulin ELISA kit (Mercodia, Uppsala, Sweden). For insulin tolerance test, 0.4 U/kg insulin (Novo Nordisk, Bagsvard, Denmark) was administered subcutaneously and blood glucose levels were measured at 0, 40, 90, and 120 min post-injection. The blood glucose curve was plotted against time and the area under the curve was calculated. For serum cytokine measurements, the fasting blood samples were collected, and the cytokine levels were measured by the use of RayBio^®^ Rat Cytokine Antibody Array G-Series 2 (RayBiotech, Norcross, GA, United States) and confirmed by ELISA (R&D Systems, Minneapolis, MN, United States). The cytokine antibody array were performed at 3 different times and the detailed results was presented in Supplementary Datasheet 1.

### Histology

Rats fed for 1, 2, 4, and 6 m were sacrificed, and liver, omentum fat and femurs were harvested and fixed with 4% paraformaldehyde (Sigma-Aldrich, St. Louis, MO, United States) followed by H&E staining. Images were captured using the image acquisition system (Olympus, Shinjuku, Japan). The percentage of fat vacuole area within hepatocytes in liver sections, diameters of adipocytes in omentum fat sections and numbers of osteoclasts in femur sections were measured separately by assessing 3 randomly selected high-power microscope fields in each slide (*n* = 5 per group).

### Rat BMSCs Isolation and Culture

The BMSCs were isolated from the femur and tibia of rats using whole bone marrow plating and subsequent passaging. The cells were cultured in BMSC growth medium consisting of DMEM (GE Healthcare, Logan, UT, United States) supplemented with 10% fetal bovine serum (FBS, Thermal Fisher Scientific, Waltham, MA, United States). The cells were maintained in an atmosphere of 5% CO2 and 95% humidity. Colonies were observed after 4–6 days and reached 80–90% confluence in approximately 12–14 days, followed by regular passaging every 3—4 days. BMSCs were isolated from 3 to 5 rats in each group.

### Cell Proliferation Assay

The 3-(4,5-dimethylthiazol-2-yl)-2,5-diphenyltetrazolium bromide (MTT, Sigma) assay was used for determination of the BMSC proliferation ability. BMSCs obtained from rats fed with the specified diets for 1, 2, 4, and 6 months were collected at the 2nd passage (P2) and seeded into 96-well plates at a density of 1 × 10^4^/well. After 12 h, which was denoted as day 0, an MTT-based assay was performed according to the manufacturer’s instructions, and the absorbance at 490 nm was recorded using a Biochrom Microplate reader. The MTT assay was performed 10 times every 24 h, and the cell number was calculated as the absorbance of the cells minus the absorbance of the medium only. The growth curves were plotted, and the population doubling time (Td) was calculated as ${\text{T}}_{\text{d}}=\frac{\Delta \,\mathrm{tLg2}}{{\text{LgN}}_{\text{t}}-{\text{LgN}}_{0}}$, where Δt denotes the days that the cells were in the exponential growth phase, N_0_ is the number of cells at the beginning of the exponential growth phase and N_t_ is the number of cells at the end of the exponential growth phase.

### Flow Cytometry Analysis

The cells were incubated with the conjugated antibodies for 30 min at 4°C, followed by two washes and analysis using a FACSAria II (BD Biosciences). The data were analyzed using FlowJo software. APC anti-rat CD29 antibody (Biolegend, 102216), FITC anti-rat CD45 antibody (Biolegend, 202205), PerCP anti-rat CD90 antibody (BD Pharmingen, 557266) and PE anti-rat CD31 antibody (BD Pharmingen, 555027) were used following manufacturer’s instructions.

### Cell Cycle and Cell Apoptosis Analysis

BMSCs at P3 were collected and processed using the Muse cell cycle kit and Muse Annexin V Dead Cell Kit (EMD Millipore, Burlington, MA, United States) following the manufacturer’s instructions. The cells were then analyzed using the Muse Cell Analyzer (Millipore).

### SA-β-Gal Staining

The β-Galactosidase (SA-β-Gal) staining Kit (Beyotime) was used for identification of senescent cells. Briefly, after fixation, 1 × 10^5^ cells/well of BMSCs at P5 were incubated in SA-β-Gal staining solution at 37°C overnight, and images were captured under a microscope. BMSCs were seeded at a density of 1 × 10^5^ cells per well of a 6-well plate.

### Oxidative Stress Measurement

For measurement of mitochondria superoxide levels, BMSCs at P5 were incubated with 5 μM MitoSOX^TM^ reagent (Thermal Fisher Scientific) for 10 min at 37°C and the images were captured after gentle washes. For reactive oxygen species (ROS) detection, BMSCs at P5 were incubated with 5mM DCF-DA (Sigma) for 10 min at 37°C and analyzed with flow cytometry (excitation/emission max = 502/523 nm).

### Differentiation of BMSCs

Osteogenic differentiation of BMSCs at P3 were induced by treating with 10 nM dexamethasone, 50 μg/ml ascorbic acid, and 10 mM β-glycerophosphate (all from Sigma-Aldrich). After incubation for 14 days, the cells were fixed with 4% paraformaldehyde and stained with 2% Alizarin Red (Sigma-Aldrich). Adipogenic differentiation of BMSCs at P3 were induced by treating with 1 × 10^–5^ M insulin, 2 × 10^–9^ M dexamethasone, 3.3 × 10^–6^ M biotin, 1.7 × 10^–5^ M pantothenic acid, 5 × 10^–6^ M rosiglitazone and 5 × 10^–4^ M 3-isobutyl 1 methylxanthine (IBMX) (all from Sigma-Aldrich). At day 10, cells were fixed and stained with Oil Red O (Sigma-Aldrich) and semi-quantified either by measuring the percentage of lipid droplet area using Image J or by eluting the dye from the cells using isopropanol and the absorbance was photometrically determined at 510 nm. Chondrogenic differentiation of BMSCs at P3 were induced by using chondrogenic differentiation kit following manufacturer’s instruction (Cyagen Biosciences, Suzhou, China). Briefly, BMSCs pellets were cultured in chondrogenic differentiation basal medium supplemented with dexamethasone, ascorbate, ITS, sodium pyruvate, proline and TGF-β3 for 21 days followed by Alcian Blue staining. To test the effects of cytokines on the osteogenic and adipogenic differentiation potentials of BMSCs, BMSCs in ND rats were isolated, and cells at P3 were treated using the same differentiation procedure but with the addition of CXCL1 or CXCL2 (R&D systems) at a dosage of 20 and 50 ng/ml, respectively.

### Microarray Analysis

The total mRNA of BMSCs (P1) from ND and HFD groups at the specified feeding time points (1, 2, and 4 months) was purified and analyzed using the Rat Genome 230 2.0 Array (Affymetrix, Santa Clara, CA, United States). GeneChip^®^ Scanner 3000 (Affymetrix) was used to collect data. The data from microarray were analyzed using Bio MAS (Molecule annotation system) 3.0 software (CapitalBio, Beijing, China). Microarray data was uploaded to GEO database and is available at series entry as GSE121036^1^.

### Real-Time PCR Analysis

Total RNA was reverse transcribed using the MessageSensor^TM^ RT Kit (Thermal Fisher Scientific) following the manufacturer’s instructions. Real-time PCR was carried out on a LightCycler 480 (Roche) using the Fast SYBR^®^ Green master mix system (Roche). The sequences of the primers are listed in Supplementary Table 2.

### Western Blotting

50μg proteins were separated by electrophoresis on an 8–10% SDS-PAGE gel, transferred to polyvinylidene fluoride membrane (Millipore) and incubated overnight at 4°C with antibodies against GAPDH (Zsbio, Beijing, China), ELMO1 and Rac1 (Abcam, Cambridge, United Kingdom). Band density was quantitated using Labworks v4.6 software (UVP, Upland, CA, United States).

### Rac1 G-LISA Activation Assay

The Rac1 activities in BMSCs were measured by using Rac G-LISA Activation Assay Biochem kit (Cytoskeleton, Denver, CO, United States) according to the manufacturer’s recommendations. The absorbance at 490 nm was measured.

### Statistical Analysis

Unless specified, all experiments were performed at least 3 independent times. Data are expressed as mean ± SEM. Differences were evaluated using Student’s *t*-test (two groups) and one-way ANOVA (three or more groups) followed by Tukey’s post hoc test. Statistical calculations were performed using Prism 5.0 (GraphPad Software). A *p*-value <0.05 was considered statistically significant.

## Results

### Serum Levels of Cytokines Were Disturbed in HFD Fed Rats

**FIGURE 1:**
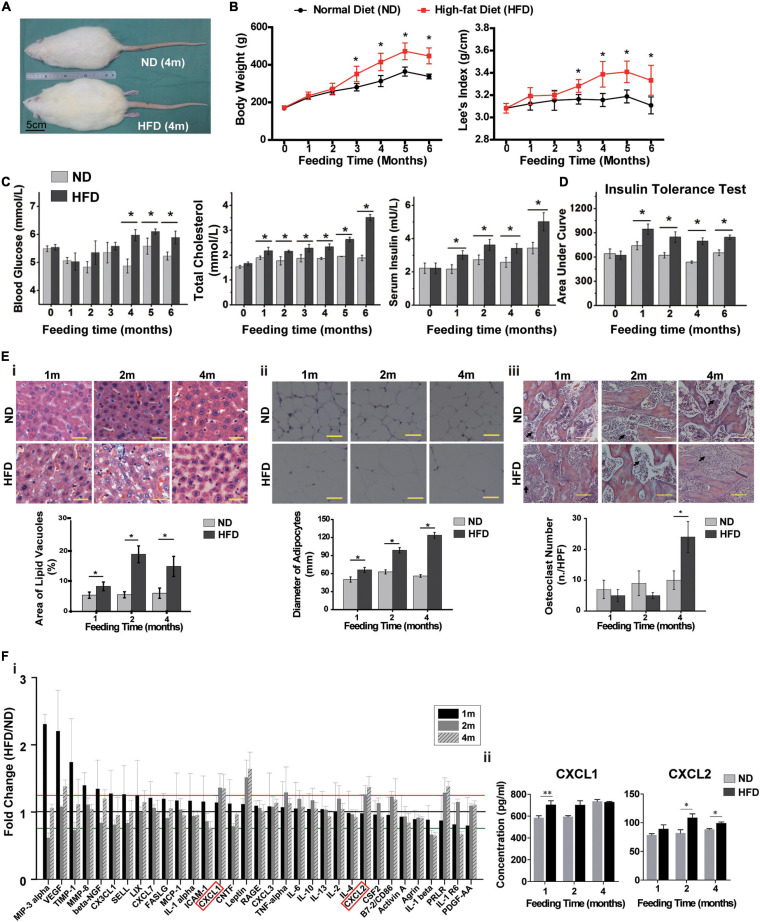
High-fat diet alters physiological state and cytokine profiles of rats. **(A)** The picture of whole rat. ND, normal diet; HFD, high-fat diet, 4 m: 4 months. Scale bar, 5 cm. **(B)** Body weight and Lee’s index, *n* = 10. **(C)** Blood glucose, total cholesterol and serum insulin levels analysis of rats. *n* = 10. **(D)** Area under curve during insulin tolerance testing. Data represent the means ± SEM of five rats. See also Supplementary Figure 1. **(E)** Representative images of H&E-stained sections and semi-quantification of liver tissues **(i)**, adipose tissues **(ii)** and bone tissues **(iii)**. The black arrows indicate the location of osteoclasts. Values are represented as mean ± SEM (*n* = 5). Scale bars, 50 μm. **(F)** Serum cytokine array analysis **(i)** and verification of CXCL1 and CXCL2 concentrations by ELISA **(ii)**. Data are presented as (mean of HFD intensity/mean of ND intensity) ± SEM (*n* = 3). Black line: 1 fold, red line: 1.2 fold, green line: 0.8 fold. Two-tailed Student’s *t*-test were used to compare two groups of means, *p < 0.05, *p < 0.05, **p < 0.01.

To assess the physiological characteristics of rats in the context of overnutrition, SD rats were fed with HFD for 6 months. HFD-fed rats showed larger body size compared to their normal diet (ND)-fed cohorts (Figure 1A). From the 3rd month onwards, the body weights and Lee’s index were significantly higher in HFD-fed rats than in ND-fed rats (Figure 1B). The blood cholesterol and serum insulin levels significantly elevated with lower sensitivity of insulin receptors after one month of HFD feeding, however, the blood glucose level remarkably increased after four months of HFD consumption (Figures 1C,D and Supplementary Figure 1B). Pathological changes in various tissues also emerged in HFD-fed rats. For instance, hepatocellular swelling (fatty degeneration of hepatocytes) and hypertrophic adipose tissue in the omentum majus were observed in the rats fed with HFD for longer than 1 month (Figures 1Ei,ii). And after 4 months of HFD feeding, a dramatic rise in the number of osteoclasts was shown in rat femur (Figure 1Eiii).

Next, we measured serum levels of rats’ cytokines and found that they were disturbed after one month of HFD feeding (Figure 1Fi). The levels of four cytokines, including Prolactin R (PRLR), Leptin, C-X-C motif chemokine ligand 1 (CXCL1) and CXCL2, showed continuous increases over 4 months and maintained 20% higher in HFD-fed rats than in ND-fed rats (*p* < 0.05). The elevated concentration of serum CXCL1 and CXCL2 in HFD rats were further verified by ELISA (Figure 1Fii). These results indicated that the metabolic and histologic changes were accompanied by a systemic inflammation in HFD-fed rats.

### BMSCs Function Were Impaired and Differentially Expressed Genes (DEGs) Were Enriched in Cytokine Signaling in HFD Fed Rats

**FIGURE 2:**
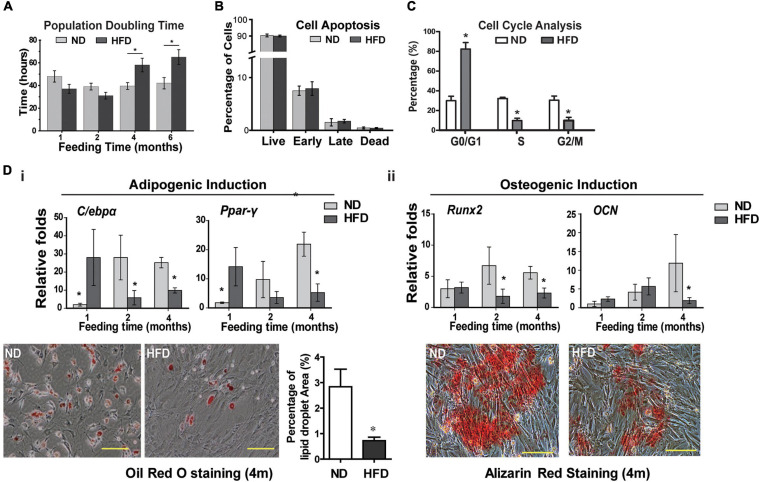
HFD feeding affects the BMSCs functions in rats **(A)** BMSCs proliferation analysis by measurement of population doubling time. **(B)** Cell apoptosis analysis. **(C)** Cell cycle analysis of rats BMSCs. **(D)** Adipogenic **(i)** and osteogenic **(ii)** induction of BMSCs. Expression of adipogenic markers (*C/ebp*α and *Ppar*-γ) and osteogenic markers (*Runx2* and *OCN*) in BMSCs was quantified with real-time PCR. Lipid droplets were visualized using Oil red O staining and the percentage of red-stained lipid droplets area were quantified by Image J. The mineralized nodules in the BMSC culture were detected using alizarin red staining. Data are represented as means ± SEM (*n* = 3), Two-tailed Student’s *t*-test were used to compare two groups of means, *p < 0.05.

Considering the contribution of BMSCs in tissue homeostasis, next, we investigated the impact of HFD on BMSCs phenotypes and transcriptome. The MSC characteristics of isolated rat BMSCs were confirmed by flow cytometry analysis (positive for MSC markers CD29 and CD90 while negative for hematopoietic marker CD45 and endothelium marker CD31, Supplementary Figure 1C), and by three-lineage differentiation assays (osteogenesis, adipogenesis and chondrogenesis, Supplementary Figure 1D). HFD consumption for 4 and 6 months markedly inhibited BMSCs proliferation, as shown by increased doubling time (Figure 2A) but did not induce BMSCs apoptosis (Figure 2B). Cell cycle analysis showed that HFD BMSCs were arrested in G1/G0 phase (Figure 2C). The expression of adipogenic markers *C/ebp*-α and *Ppar*-γ in adipogenic induced BMSCs were significantly augmented in the HFD group compared with the ND group after 1 month of feeding, but rapidly decreased after 2 and 4 months with reduced numbers of lipid droplets (Figure 2Di). Meanwhile, the decreased osteogenic differentiation potential of BMSCs was also observed after 2 and 4 months of HFD feedings, as shown by lower expression of osteogenic markers *Runx2* and *OCN* and fewer numbers of alizarin red-stained mineralized nodules after osteogenic induction (Figure 2Dii). Collectively, these results showed that HFD has deleterious effects on BMSCs stemness *in vivo*.

**FIGURE 3:**
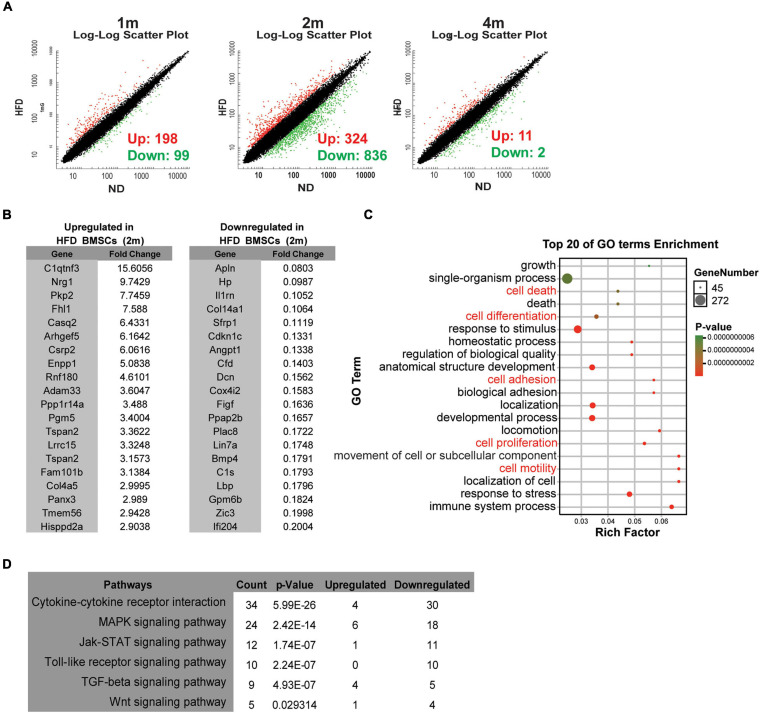
HFD feeding alters the transcription profiles of BMSCs *in vivo* **(A)** Microarray analysis of BMSCs isolated from ND rats and HFD rats. Scatter plots of the BMSCs from rats in specified diets for 1 month (1 m), 2 months (2 m) and 4 months (4 m). **(B)** Lists of the top 20 up-regulated and down-regulated genes in HFD BMSCs compared with ND BMSCs. **(C)** The bubble chart shows Gene Ontology (GO) enrichment analysis in HFD BMSCs. **(D)** The enriched KEGG pathways in HFD BMSCs. *n* = 3.

The transcriptome of BMSCs by microarray analysis showed that the expressions of 297 genes in BMSCs were significantly changed with 198 genes up-regulated and 99 down-regulated after 1 month of HFD feeding. Though the number of DEGs increased to 1,160 at 2 months of feeding, after 4 months of HFD, the number of DEGs dramatically decreased with only 11 up-regulated and 2 down-regulated (Figure 3A). Therefore, we listed genes with the most significant changes (top 20) in HFD BMSCs at 2 months in Figure 3B, and 16 of them were verified by real-time PCR assay (Supplementary Figure 2). DEGs were enriched in genes involved in cell growth, death, differentiation, adhesion, proliferation and motility, etc (Figure 3C). The KEGG pathway analysis identified the cytokine signaling as the most significant pathways, including cytokine-cytokine receptor interaction and its downstream signalings including MAPK signaling and JAK-STAT signaling (Figure 3D).

### CXCL2 Suppressed the Adipogenic Potential of BMSCs Through ELMO1/Rac1 Signaling *in vitro*

**FIGURE 4:**
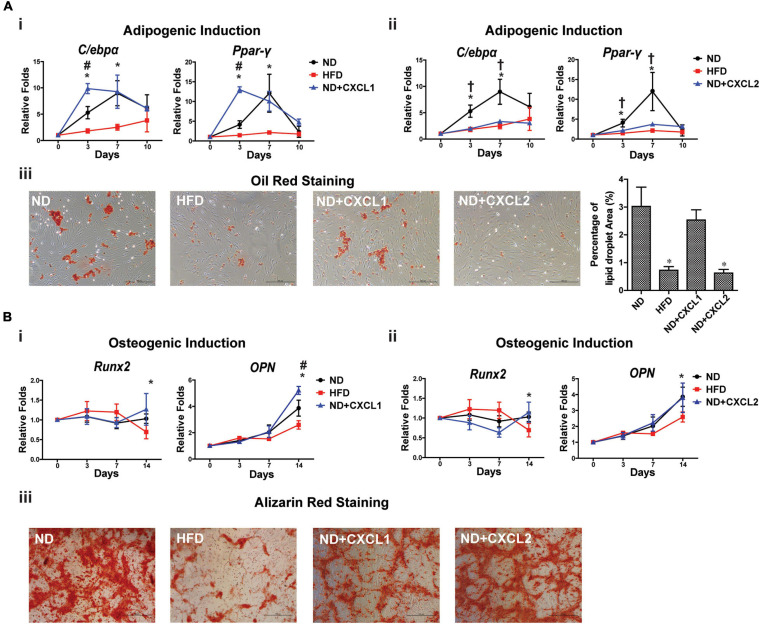
CXCL2 affects the differentiation potentials of BMSCs *in vitro*. **(A)** Effects of CXCL1 (20 ng/ml) **(i)** and CXCL2 (50 ng/ml) **(ii)** on the expressions of adipogenic markers in BMSCs isolated from ND-fed rat during differentiation *in vitro*. Lipid droplets were visualized using Oil red O staining and the percentage of red-stained lipid droplets area were quantified by Image J **(iii)**. **(B)** Effects of CXCL1 (20 ng/ml) **(i)** and CXCL2 (50 ng/ml) **(ii)** on the expressions of osteogenic markers in BMSCs isolated from ND-fed rat during osteogenic differentiation *in vitro*. The mineralized nodules in the BMSC culture were detected using alizarin red staining **(iii)**. Data are represented as mean ± SEM (*n* = 4). Significance was calculated by one-way ANOVA followed by Tukey’s post hoc test, *p < 0.05 (ND versus HFD), ^#^*p* < 0.05 (ND versus ND + CXCL1), ^†^*p* < 0.05 (ND versus ND + CXCL2).

According to the transcriptome changes of BMSCs, we would like to determine whether an increase of serum cytokines CXCL1 and CXCL2 was correlated with impaired BMSCs stemness. We tracked differentiation potentials of ND BMSCs following CXCL1 and CXCL2 treatment *in vitro*. In adipogenic induced ND BMSCs (Figure 4A), CXCL1 treatment resulted in a transient increase of *C/ebp*-α and *Ppar*-γ expressions, in contrast, CXCL2 dramatically reduced the expressions of *C/ebp-*α and *Ppar*-γ with significant reduction of the lipid droplet formation. During osteogenic differentiation, addition of CXCL1 did not cause significant differences in *Runx2* expression but instead enhanced *OPN* expression by day 14 (Figure 4Bi), while addition of CXCL2 did not alter the *Runx2* and *OPN* expressions in ND BMSCs (Figure 4Bii). Alizarin red staining also demonstrated that CXCL1 and CXCL2 did not significantly affect the mineralized nodules formation of osteogenic-induced ND BMSCs (Figure 4Biii). These results suggest that the elevation of CXCL2 levels could decrease the adipogenic differentiation ability of BMSCs.

**FIGURE 5:**
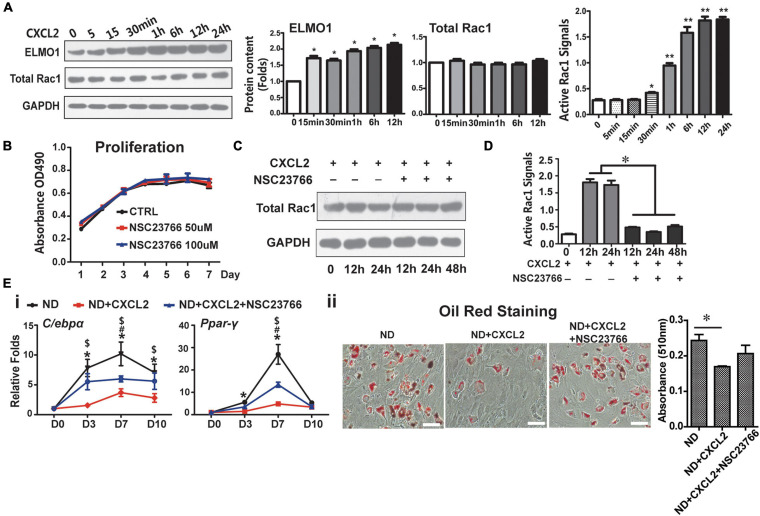
CXCL2 modulates functions of BMSCs through ELMO/Rac1-mediated signaling. **(A)** Effects of CXCL2 on the protein levels of ELMO1 and total Rac1 (as measured by western blot analysis) and on the Rac1 activity (as assessed by Rac1 G-LISA assay) in BMSCs. Significance was calculated by student’s *t*-test, *p < 0.05, **p < 0.01, *n* = 3. **(B–D)** Effects of Rac1 inhibitor NSG23766 on the proliferation **(B)**, Rac1 level **(C)** and Rac1 activity **(D)** of 6N BMSCs with or without CXCL2 treatment. Significance was calculated by student’s *t*-test, *p < 0.05, *n* = 3. **(E)** Effects of NSG23766 on adipogenic differentiation of 6N BMSCs with CXCL2 treatment. Expression of adipogenic differentiation markers was quantified with real-time PCR analysis **(i)** the lipid droplet formation was detected using oil red staining **(ii)**. Significance was calculated by one-way ANOVA followed by Tukey’s post hoc test, *p < 0.05 (6N and 6N+CXCL2), ^#^*p* < 0.05 (6N and 6H + CXCL2 + NSC23766), ^$^*p* < 0.05 (6N + CXCL2 and 6H + CXCL2 + NSC23766), *n* = 3. Scale bars, 50 μm.

CXCL2 is a well-known potent neutrophil chemoattractant and mainly functions through CXCR2 (Al-Alwan et al., 2013). The molecular detail underlying CXCL2/CXCR2 regulation on MSC functions is unknown. It has been previously shown that IL-8, also known as CXCL8, binds to CXCR2 and modulates cell migration and invasion by activating the small RhoGTPase Rac1 in an ELMO1-dependent manner (Zhang B. et al., 2015), and cell shape-dependent RAC1 activation also plays important roles in the differentiation and lineage commitment of human MSCs (Gao et al., 2010). These findings led us to examine the levels of ELMO1 and activated Rac1 in BMSCs in response to CXCL2 treatment. The addition of CXCL2 dramatically increased the levels of ELMO1 protein and activated Rac1 in BMSCs in a time-dependent manner, without altering the total Rac1 protein level (Figure 5A). Use of NSC23766, a specific inhibitor of Rac1 activation, did not influence cell proliferation (Figure 5B) and the total Rac1 level (Figure 5C), but significantly suppressed CXCL2-induced Rac1 activation (Figure 5D). It also partially abrogated CXCL2-induced reduction of adipogenic potential of BMSCs (Figure 5E). These results indicate that CXCL2 affected the BMSCs stemness and inhibited the adipogenic differentiation potential of BMSCs through ELMO1/Rac1-mediated signaling.

### CXCL2 Promotes Senescence and Migration of BMSCs *in vitro*

**FIGURE 6:**
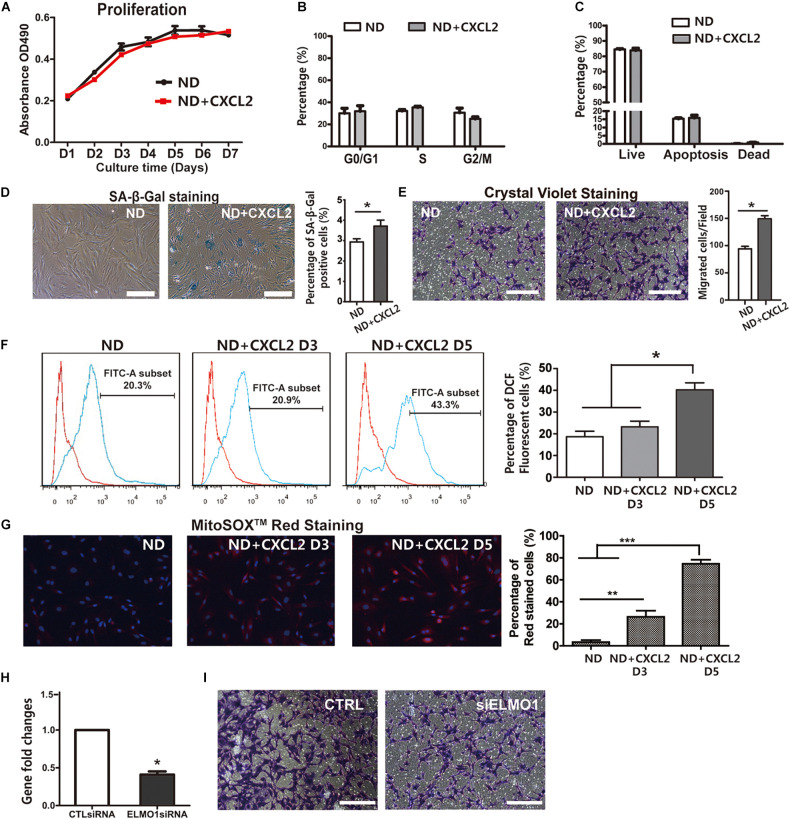
CXCL2 impairs function and promotes cellular senescence and migration of BMSCs *in vitro*. **(A–C)** Proliferation **(A)**, cell cycle **(B)** and the rate of apoptosis **(C)** of 6N BMSCs in response to CXCL2 treatment. **(D)** Characterization of the effects of CXCL2 on cellular senescence of 6N BMSCs using SA-β-Gal staining. **(E)** Effects of CXCL2 on the migration of 6N BMSCs as assessed with crystal violet staining. Significance was calculated by student’s *t*-test, *p < 0.05, *n* = 5. Scale bars, 50 μm. **(F,G)** Measurement of ROS production of 6N BMSCs using DCF-DA staining **(F)** and MitoSOX-Red staining **(G)**. Significance was calculated by student’s *t*-test, *p < 0.05, **p < 0.01, ***p < 0.001, *n* = 5. **(H–I)** Reduced migration of BMSCs after treatment with siRNA-mediated Knockdown of ELMO1 (siELMO1). The mRNA expression level of ELMO1 in 6N BMSCs was assessed by real-time PCR **(H)** and the migration abilities of BMSCs were detected with crystal violet staining **(I)**. Significance was calculated by student’s *t*-test, *p < 0.05, *n* = 3. Scale bars, 50 μm.

To determine the role of CXCL2 elevation in BMSCs dysfunction other than impaired differentiation potentials during HFD feeding, ND BMSCs were treated with CXCL2 *in vitro*. The addition of CXCL2 to BMSCs did not exert a significant effect on cell proliferation (Figure 6A), cell cycle progression (Figure 6B) or apoptosis (Figure 6C), whereas it markedly promoted the cell senescence (Figure 6D) and migration of BMSCs (Figure 6E). Oxidative stress refers to the excessive production of reactive oxygen species (ROS) and is a well-established trigger of senescence (Barbouti et al., 2020). Mitochondria are important sites for ROS production (Kong et al., 2014). A significant increase in both ROS level (Figure 6F) and superoxide production of mitochondria (Figure 6G) were observed in BMSCs after CXCL2 treatment in a time-dependent manner, indicating that CXCL2-induced BMSC senescence was attributed by the occurrence of oxidative stress. We next assessed whether ELMO1/RAC1 signaling was involved in the regulation of BMSC migration. As expected, suppression of ELMO1 by siRNA significantly reduced the migration of BMSCs (Figures 6H,I). Collectively, these data demonstrated that the elevated CXCL2 level could directly induce BMSCs senescence by causing increased ROS production in mitochondria and promote BMSC migration via ELMO1-dependent pathway.

### Serum Cytokine Levels and BMSC Functions After 2 Months of HFD Feeding Cannot Be Fully Rescued by Diet Correction

**FIGURE 7:**
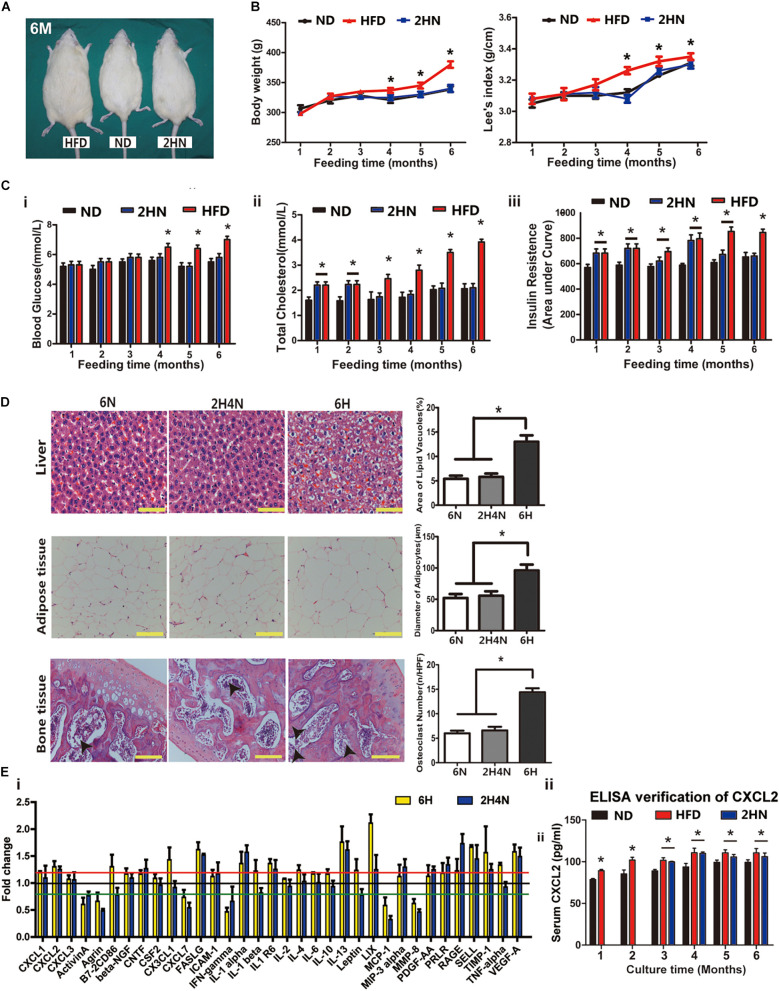
Dietary intervention restores the physio-biochemical characteristics of the HD rats while the serum cytokine profiles remain changed. **(A)** Sizes of representative rats fed for 6 months in ND, HFD and 2HN groups. Scale bar, 5 cm. **(B)** Body weight and Lee’s index of rats in ND, HFD and 2HN groups over 6 months of feeding (*n* = 10). Significance was calculated by one-way ANOVA followed by Tukey’s post hoc test, *p < 0.05 (ND and 2HN versus HFD). **(C)** Blood glucose **(i)**, total cholesterol level **(ii)**, and insulin tolerance test **(iii)** of rats (*n* = 10) in ND, HFD and 2HN groups over 6 months of feedings. Significance was calculated by one-way ANOVA followed by Tukey’s post hoc test, *p < 0.05. **(D)** Histological analysis of liver, adipose and bone tissues of rats in 6N, 6H, and 2H4N groups. Values are represented as mean ± SEM. Scale bars, 50 μm. **(E)** Serum cytokine profiles of rats in 6H and 2H4N groups, presented as the mean of fold changes ± SEM (*n* = 3) in comparison with 6N rats **(i)**. ELISA verification for serum CXCL2 levels in rats over 6 months of feedings **(ii)**. Significance was calculated by student’s *t*-test, *p < 0.05.

To detect whether the physiological defects and BMSC functions of rats could be rescued through diet correction, we shifted the diet to ND after 2 months of HFD feeding and termed the rats as 2HN, in which we previously showed that the transcriptome of BMSCs dramatically changed without the significant alteration in body weights and Lee’s index. Rats fed with either ND or HFD continuously for 6 months were termed as 6N and 6H respectively, while the rats with 4 months of diet-correction were termed as 2H4N. The body size (Figure 7A) weight gain (Figure 7B) and fasting blood glucose levels (Figure 7Ci) in 2HN rats were comparable with those in ND rats which were significantly smaller/lower than those in HFD rats. Additionally, one and four months of dietary intervention could restore the fasting cholesterol levels and insulin tolerance in 2HN rats respectively (Figures 7Cii,iii). Histological analysis revealed that HFD induced pathological changes in liver, adipose and bone tissues were not observed in 2H4N rats (Figure 7D). These findings suggested that diet intervention can restore the metabolic parameters and histopathology of liver, adipose and bone in 2HN rats to near normal state.

**FIGURE 8:**
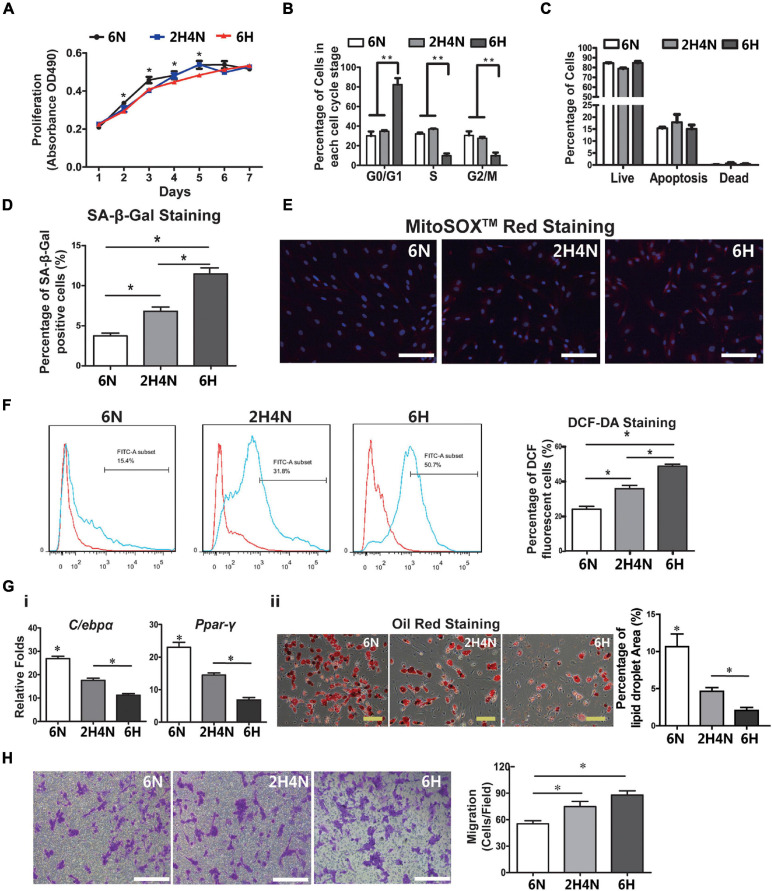
BMSC functions cannot be reversed by dietary intervention. **(A–C)** Cell proliferation **(A)**, cell cycle **(B)** and apoptosis analysis **(C)** of BMSCs in 6N, 6H and 2H4N groups. **(D)** Percentage of SA-β-Gal-positive senescent cells of BMSCs in 6N, 6H and 2H4N groups. **(E,F)** The MitoSOX^TM^ Red staining **(E)** and the measurement of reactive oxygen species (ROS) levels as judged by DCF fluorescence analyzed by flow cytometry after DCF-DA staining **(F)** of BMSCs in 6N, 6H and 2H4N groups, *n* = 5. **(G)** Expression of adipogenic markers **(i)** and oil-red staining **(ii)** of adipogenic-induced BMSCs in 6N, 6H and 2H4N groups. **(H)** and crystal violet staining of BMSCs in the 6N, 6H and 2H4N groups, *n* = 5. Significance was calculated by one-way ANOVA followed by Tukey’s post hoc test, *p < 0.05, **p < 0.01. Scale bars, 50 μm.

As to serum cytokines in 2H4N rats, a subset of up-regulated cytokines including TNF-alpha were restored to the similar levels of 6N rats, whereas CXCL2, PRLR, LIX, IL-1α, IL-1R6, IL-13, FASLG, VEGF-A, SELL and TIMP1 remained up-regulated (Figure 7Ei), suggesting that dietary intervention could not fully reverse the perturbed cytokine levels of HFD-fed rats. Specifically, CXCL2 level was significantly upregulated in the HFD and 2HN groups over the entire experimental period, as verified with ELISA (Figure 7Eii). The analysis of BMSC characteristics showed the reduced proliferation and G0/G1-phase arrest in 6H rats, while 2H4N BMSCs shared comparable proliferation and cell-cycle progression with 6N BMSCs (Figures 8A,B). We also observed that the fraction of senescent cells and ROS levels in 2H4N BMSCs were remarkably lower than in the 6H group, although they remained higher than in the 6N BMSCs (Figures 8D–F). Additionally, adipogenic potential of 2H4N BMSCs were notably increased than those in the 6H group and remained lower than those in the 6N group, as shown by the expressions of *C/ebp-*α, *Ppar*-γ (Figure 8Gi) and numbers of lipid droplets (Figure 8Gii). BMSCs also exhibited higher migration capacity in 2H4N group than in 6N group but lower than in the 6H group (Figure 8H). Collectively, these data indicate that diet correction can ameliorate but not fully reverse the serum cytokine disturbance (especially CXCL2 elevation) and BMSCs dysfunctions induced by HFD.

### Serum CXCL2 Level Indicates Dysfunctional BMSC in HFD Fed Rats

**FIGURE 9:**
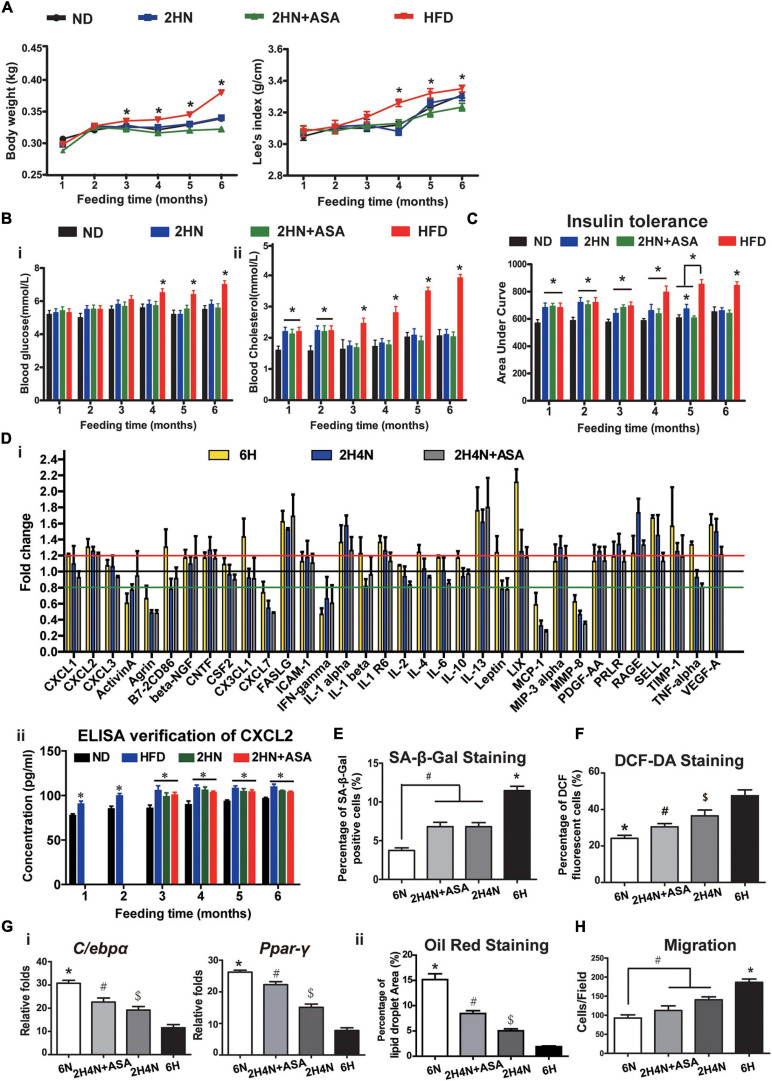
Aspirin administration promotes recovery of insulin tolerance and ameliorates the cytokine profiles in rats, while partially reversing CXCL2 level and BMSC functions. **(A–C)** Body weight gain and Lee’s index **(A)**, blood glucose and cholesterol levels [**(B)** *n* = 10] and insulin tolerance [**(C)** *n* = 5] of rats in the ND, HFD, 2HN and 2HN+ASA groups over 6 months of feedings. **(D)** Serum cytokine profiles of rats in the 6H, 2H4N and 2H4N+ASA groups **(i)**. Data are presented as the mean of fold changes ± SEM (*n* = 3) in comparison with 6N rats. ELISA was applied to verify the serum CXCL2 levels **(ii)**, *n* = 3. Significance was calculated by one-way ANOVA followed by Tukey’s post hoc test, *p < 0.05. **(E)** SA-β-Gal staining of senescent BMSCs. *p < 0.05 (6H versus another 3 groups), ^#^*p* < 0.05 (6N versus 2H4N and 2H4N+ASA). **(F)** DCF-DA staining of BMSCs. *p < 0.05 (6N versus another 3 groups), ^#^*p* < 0.05 (2H4N+ASA versus 2H4N and 6H), ^$^*p* < 0.05 (2H4N versus 6H). **(G)** Adipogenic differentiation potential of BMSCs were assessed by the expressions of adipogenic markers **(i)** in BMSCs and quantification of percentage of oil red-stained lipid droplet area **(ii)**. Significance was calculated by one-way ANOVA followed by Tukey’s post hoc test, *p < 0.05 (6H versus another 3 groups), ^#^*p* < 0.05 (2H4N+ASA versus 2H4N and 6H), ^$^*p* < 0.05 (2H4N versus 6H). **(H)** Migration capacities of BMSCs in 6N, 6H, 2H4N and 2H4N+ASA groups. Significance was calculated by one-way ANOVA followed by Tukey’s post hoc test, ^#^*p* < 0.05 (2H4N and 2H4N+ASA versus 6N), *p < 0.05 (6H versus another 3 groups). *n* = 5.

To further correct serum cytokine disturbances and BMSC dysfunctions, we applied aspirin treatment in addition to diet correction in 2HN rats (hereafter termed as 2HN+ASA). There were similar changes in the weight gain, fasting blood glucose and cholesterol levels between 2HN+ASA rats and 2HN rats (Figures 9A,B), whereas the recovery of insulin tolerance was accelerated to normal level after 3 months of aspirin treatment in 2HN+ASA rats, instead of 4 months diet intervention in 2HN rats (Figure 9C). The serum levels of CXCL1, Leptin and PRLR in 2H4N+ASA rats returned to similar levels in 6N rats, however, CXCL2 continuously remained to be higher (20%) than that in 6N rats and the significance was confirmed by ELISA (Figure 9D).

Moreover, compared to 2H4N group, additional aspirin treatment failed to decrease the number of senescent BMSCs (Figure 9E and Supplementary Figure 3A) but reduced oxidative stress levels in the 2H4N+ASA group (Figure 9F and Supplementary Figures 3B,C). The adipogenic potential of BMSCs from the 2H4N+ASA group was enhanced but still lower than that of BMSCs from the 6N group (Figure 9G and Supplementary Figure 3D), the migration capacity of BMSCs was mildly corrected as well (Figure 9H and Supplementary Figure 3E). These data indicate that anti-inflammatory treatment, the use of aspirin with diet intervention, can further ameliorate the perturbed cytokine levels but is incapable of restoring the circulating CXCL2 level which might be tightly correlated with the HFD-induced BMSC dysfunctions.

## Discussion

Diet-induced physiological cues including both local and circulating signals can influence stem cell behaviors (Nakada et al., 2011). Here, we show that the continuously elevated serum CXCL2 level is closely correlated with the dysfunction of BMSCs in HFD-fed rats *in vivo* and CXCL2 treatment can directly contribute to the dysfunction of BMSCs through ELMO/Rac1-mediated signaling *in vitro*. Importantly, although glycolipid metabolism indicators can be corrected, the CXCL2 elevation and BMSC dysfunctions cannot be fully rescued by diet correction and anti-inflammatory aspirin treatment, indicating the long-lasting deleterious effects of HFD on serum CXCL2 levels and BMSC functions. Therefore, we speculate that serum CXCL2 level might represent a serum indicator reflecting the impairment of BMSCs induced by HFD.

In our study, cholesterol metabolism and insulin tolerance are disturbed more rapidly than the occurrence of hyperglycemia and body overweight in response to HFD. A population-based study also reported that cholesterol metabolism is altered already in human subjects with impaired fasting glucose (Gylling et al., 2010). Moreover, our results showed that circulating cytokine profiles and BMSCs characteristics were already altered in normoglycemic rats soon after 1 month of HFD feeding, accompanied with the pathological changes (fatty infiltration of the liver and enlarged adipocytes). Therefore, it is reasonable to speculate that cytokine profiles might represent an early indicator reflecting the onset of HFD-induced metabolic disorders and the dysfunctions of BMSCs in subjects. We further demonstrated that the continuous elevation of CXCL2 could affect the functions of BMSCs. CXCL2, also known as macrophage inflammatory protein 2 (MIP-2), belongs to the CXC family of chemokines eliciting neutrophil accumulation at the site of inflammation via the PI3Kγ and Akt/PKB-dependent pathways (Iida et al., 1992; Lehman et al., 2006). CXCL2 expression has been shown to be induced by pro-inflammatory cytokines, such as IL-1 and TNF-α, both *in vitro* (Liu et al., 2000; De Plaen et al., 2006) and *in vivo* (Calkins et al., 2002; Li et al., 2014). In our study, circulating levels of TNF-α and IL-1α in HFD rats were up-regulated even after the diet correction for 4 months and therefore might be responsible for the sustained CXCL2 production.

From a mechanistic perspective, we demonstrate *in vitro* that CXCL2 can affect the functions of BMSCs directly through ELMO1/Rac1 signaling. Rac1, along with RhoA and Cdc42, is one of the most prominent Rho family GTPases and plays a key role in the induction of actin lamellipodia, cell-matrix adhesion and cell anoikis (Guo et al., 2006). Rac1 activation is mediated by evolutionarily conserved complexes consisting of ELMO1 (engulfment and cell motility 1) and dedicator of cytokinesis 180 (Dock180) (Wang et al., 2016). Rac1 activity directs a switch between well-spread smooth muscle cells and rounded chondrogenic MSCs in the presence of TGFβ3 through N-cadherin (Gao et al., 2010), while inhibition of Rac1 activity promotes BMP-2-induced MSC osteogenesis (Onishi et al., 2013). The work by Gao et al. showed that under pro-adipogenic conditions, the expression of constitutively active Rac1 inhibits adipogenesis in human MSCs (Gao et al., 2010). In addition, CXCR2 (IL-8RB), the receptor of CXCL2, is reportedly present on the surface of MSCs and plays an important role in MSC migration (Halpern et al., 2011). Our results further revealed that ELMO1/Rac1 activation inhibited the adipogenesis and promoted migration of rat BMSCs in the presence of CXCL2.

We also demonstrated that HFD-induced elevation of CXCL2 is responsible for BMSCs senescence by enhancing production of mitochondrial ROS. Functional and regenerative capacities of MSCs are known to decline with senescence, while the underlying mechanisms that control MSC senescence are not well understood. The work by Zhang et al. showed that during the aging process in human and mice, mitochondrial Ndufs6 expression was significantly decreased in aged BMSCs and accelerated BMSC senescence (Zhang et al., 2020). However, based on our microarray results, we did not detect significant depressed expression of mitochondrial Ndufs6 in BMSCs isolated from rats fed with HFD for up-to 12 months (Supplementary Figure 4A). The inconsistency between our findings and Zhang’s report may be due to the different causes of BMSCs senescence. In Zhang’s report, BMSC senescence was occurred during aging process, whereas the BMSCs senescence in our results was induced by HFD feeding. It has also been reported that FGF21 is a responsive serum marker of mitochondrial stress in human (Suomalainen et al., 2011) and depletion of FGF21 and suppressed FGF21 signaling enhanced production of mitochondrial ROS and increased the senescence of early-passage human MSCs (Li et al., 2019). However, our microarray data showed that HFD feeding for up to 12 months (12M) did not significantly alter the expressions of Fgf21 signaling genes, including Fgfr1 (FGF21 receptor) and Atf4 (transcription factor that positively regulate FGF21) in BMSCs, while the expression of Slc2a1 (FGF21 target) of BMSCs were only transiently depressed in rats fed with HFD for 2 months (Supplementary Figures 4B–D). Collectively our findings suggested that FGF21 signaling was not suppressed in BMSCs in HFD-fed rats. This discrepancy is most likely due to a species difference between humans and rats, for instance, it has been reported that species variation was present in the mechanisms of MSC-mediated immunosuppression (Ren et al., 2009).

Another important function of MSC in regenerative medicine is paracrine function that is tightly regulated by telomere associated protein RAP1/NF-κb signaling (Zhang Y. et al., 2015; Ding et al., 2018). However, based on our microarray results, no significant changes of RAP1 expression or NF-kB signaling were observed in HFD BMSCs compared with ND BMSCs (Supplementary Figure 4E). Nevertheless, KEGG pathway analysis of microarray data showed that the DEG in HFD BMSCs were enriched in cytokines/cytokine receptor interaction and 30 cytokine/cytokine receptor-encoding genes were significantly decreased while 4 genes were significantly up-regulated. These findings suggested that the paracrine function of BMSCs was also impaired in HFD-treated rats.

Effective preventive approaches of HFD-induced diseases include lifestyle changes, primarily weight loss, diet, and exercise (Kaur, 2014). However, insulin sensitivity or the endothelial dysfunction induced by HFD cannot be improved after returning to a normal diet, suggesting the existence of metabolic memory in the HFD-induced model (Tallapragada et al., 2015; Testa et al., 2017). Although metabolic memory is defined as the persistent deleterious effects of initial hyperglycemia on the development of diabetic vascular complications even after glucose normalization (Tallapragada et al., 2015; Luna et al., 2016), our data provide the first evidence for the existence of metabolic memory with altered BMSC functions and cytokine CXCL2 levels after the diet correction. Oxidative stress, non-enzymatic glycosylation of proteins, epigenetic changes, and chronic inflammation have been proposed to contribute to the underlying pathophysiologic mechanisms of metabolic memory (Testa et al., 2017). In the current study, the metabolic consequences of a HFD on BMSCs were directly linked to the level of CXCL2, and they could be partially reversed by aspirin treatment, reinforcing the idea that prolonged inflammation mediate metabolic memory in HFD-fed rats.

Since HFD-induced disorders have features of chronic inflammation and a hypercoagulable state, the use of aspirin as adjunctive therapy for patients with metabolic syndrome has been emphasized (Shields and Hennekens, 2004; Group et al., 2018; Norgard, 2018). The basis of the therapeutic indications for aspirin is predicated on its vasodilatory, antithrombotic, and anti-inflammatory effects (Gao et al., 2009; Gardner et al., 2009; Patti et al., 2019). Aspirin administration also has been shown to improve HFD-induced hyperinsulinemia and hyperlipidemia and to attenuate insulin resistance in animal models (Carvalho-Filho et al., 2009; Lin et al., 2014). The effects of aspirin on lowering blood glucose level and improving insulin resistance have also been noted in a number of small-scale clinical and animal studies (Prince et al., 1981; Yuan et al., 2001; Sun et al., 2011; Amiri et al., 2015). The mechanism by which aspirin influences glucose metabolism is unclear, but it likely involves anti-inflammatory pathways. For instance, aspirin may enhance insulin sensitivity by inhibiting TNF-α production and protecting IRS-1 from TNF-α-induced serine phosphorylation (Gao et al., 2003; Sun et al., 2011). Additional studies showed that aspirin reverses hyperglycemia, hyperinsulinemia, and dyslipidemia by inhibiting the activity of NF-κB and its upstream activator IKK-β in obese rodents (Yuan et al., 2001; Sun et al., 2011; Bako et al., 2019). Consistently, in our study, we observed improved insulin sensitivity of the rats that received concomitant aspirin treatment and dietary intervention. Our results also showed that aspirin treatment led to a significant reduction in pro-inflammatory cytokines including TNF-α and IL-6 which may activate NF-κB signaling.

In addition, although MSCs are widely present in the stromal fraction of many adult tissues, in particular from the bone marrow and adipose, batch-to-batch variations, variable of stem cell senescence and proliferative potency of MSCs derived from different individuals/donors severely affect their uses in clinical applications and the accuracy in MSC studies (Kimbrel et al., 2014). Pluripotent stem cells-derived MSC (PSC-MSC) with consistent quality and highly proliferative may provide another putative cellular source overcome many limitations of adult MSC (Zhang et al., 2012; Bloor et al., 2020). In our study, we demonstrated that elevated CXCL2 could impair the functions and promote senescence of BMSCs *in vitro*. In future study, PSC-MSCs will be a more recommended cell source for the further confirmation and investigations of the effects and the underlying mechanisms of CXCL2 on MSCs.

In conclusion, our study reveals the intricate link among high-fat intake, chronic inflammation and BMSC dysfunction. We have identified that elevated serum CXCL2 might be used as a serum indicator for the deregulated inflammatory tone and can predict the altered functions of BMSCs induced by HFD. Moreover, aspirin treatment with dietary intervention significantly ameliorates the behaviors of BMSCs and serum CXCL2 level in our study, supporting the potential use of longer duration of aspirin treatment to protect BMSCs from sustained HFD-induced inflammation. Our findings will contribute to a deeper understanding of the role of circulating cytokines, especially CXCL2, in the controlling of BMSCs functions in response to the metabolic stress.

## Data Availability Statement

The original contributions presented in the study are publicly available. This data can be found here: https://www.ncbi.nlm.nih.gov/geo/query/acc.cgi?acc=GSE121036.

## Ethics Statement

The animal study was reviewed and approved by Institutional Animal Care and Use Committee of Plastic Surgery Hospital (Institute).

## Author Contributions

RX administrated and conceptualized the research studies. JB, QL, and XF performed the experiments and wrote the original draft. JB, QL, XF, LC, ZY, and TL collected and analyzed the data. JB, QL, XF, XY, LY, XL, and QW interpreted the data. XF and RX reviewed and edited the draft, and acquired funding. All authors read and approved the content of the manuscript.

## Conflict of Interest

The authors declare that the research was conducted in the absence of any commercial or financial relationships that could be construed as a potential conflict of interest.

## Footnotes

1 https://www.ncbi.nlm.nih.gov/geo/query/acc.cgi?acc=GSE121036

## Abbreviations

ADSCs: adipose-derived mesenchymal stem cells
ASA: acetylsalicylic acid
BMSCs: bone marrow mesenchymal stem cells
CVD: cardiovascular diseases
FBS: fetal bovine serum
HFD: high-fat diet
LOI: Lee obesity index
MSC: mesenchymal stem cells
ND: normal diet
PCR: polymerase chain reaction
ROS: reactive oxidative species
SA-β-Gal: senescence-associated β-Galactosidase
2HN: Rats fed with high fat diet for 2 months followed by normal diet feeding
2H4N: Rats fed with high fat diet for 2 months followed by 4 months of normal diet feeding
6H: Rats fed with high fat diet for 6 months
6N: Rats fed with normal diet for 6 months.
