# Supplementary material for: CXCL2 Impairs Functions of Bone Marrow Mesenchymal Stem Cells and Can Serve as a Serum Marker in High-Fat Diet-Fed Rats
Source: Front Cell Dev Biol. 2021 Jul 13;9:687942. doi: 10.3389/fcell.2021.687942 (PMC8315099; doi:10.3389/fcell.2021.687942)
Supplement: Supplementary file 1 [file Data_Sheet_1.pdf]

Supplementary Figures

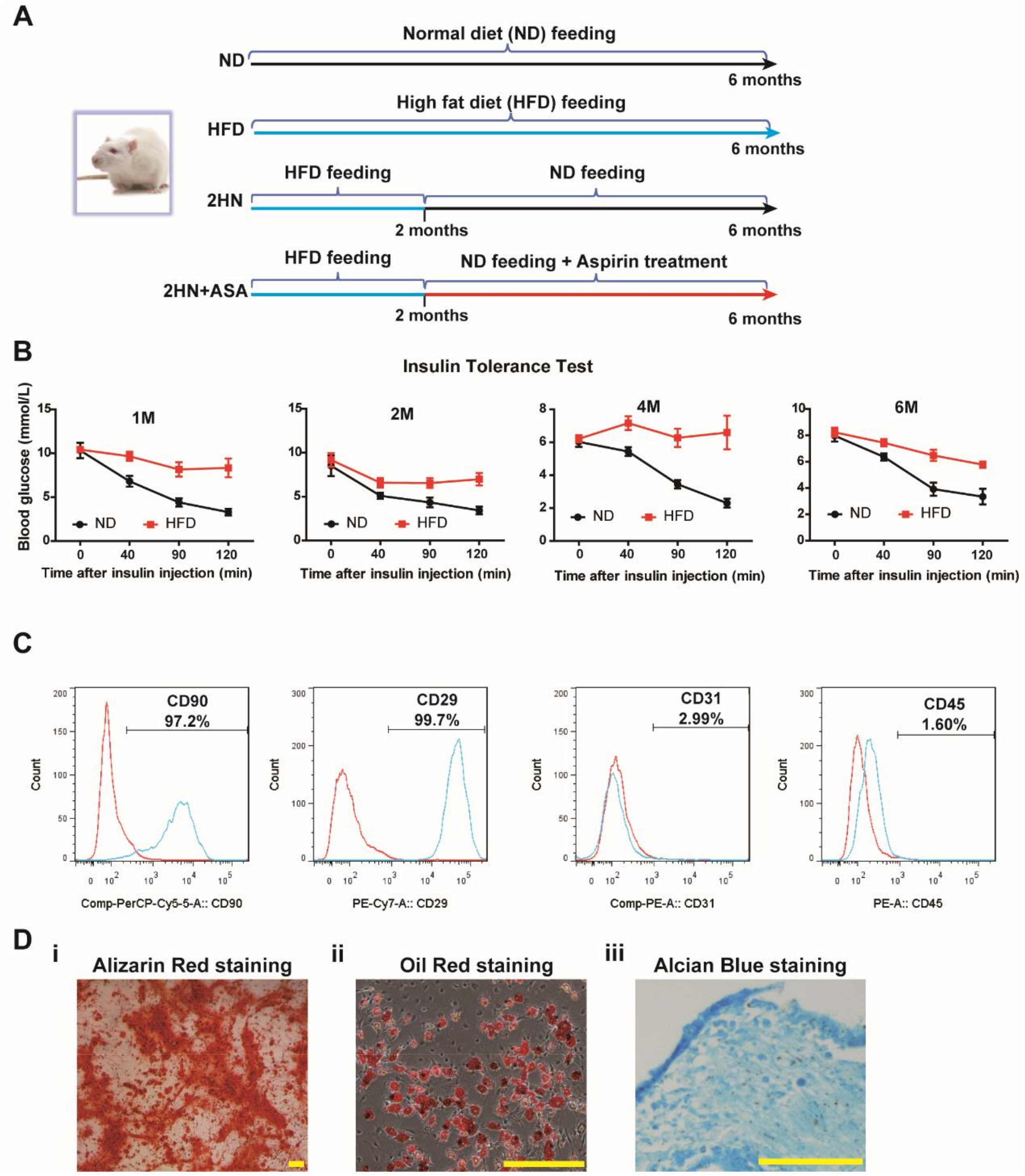

Supplementary Figure 1. Schematic demonstration of animal experiment and the characterization of BMSCs. (A) Schematic demonstration of animal experiment and grouping. (**B**) Blood glucose concentration of rats during insulin tolerance test. (**C**) Analysis of MSC phenotypic markers of rat BMSCs (positive for CD90 and CD29 and negative for CD31 and CD45 expression) by flow cytometry, n=3. **(D)** Analysis of

osteogenic, adipogenic and chondrogenic differentiation potentials of BMSCs. BMSCs were cultured in osteogenic induction medium for 14 days followed by alizarin red staining for detection of mineral nodules (i). Adipogenic induction of BMSCs were carried out for 14 days followed by Oil red staining for visualization of lipid droplet (ii). BMSCs were cultured under chondrogenic conditions for 21 days and then assessed by Alcian blue staining (iii). Scale bar: 100μm.

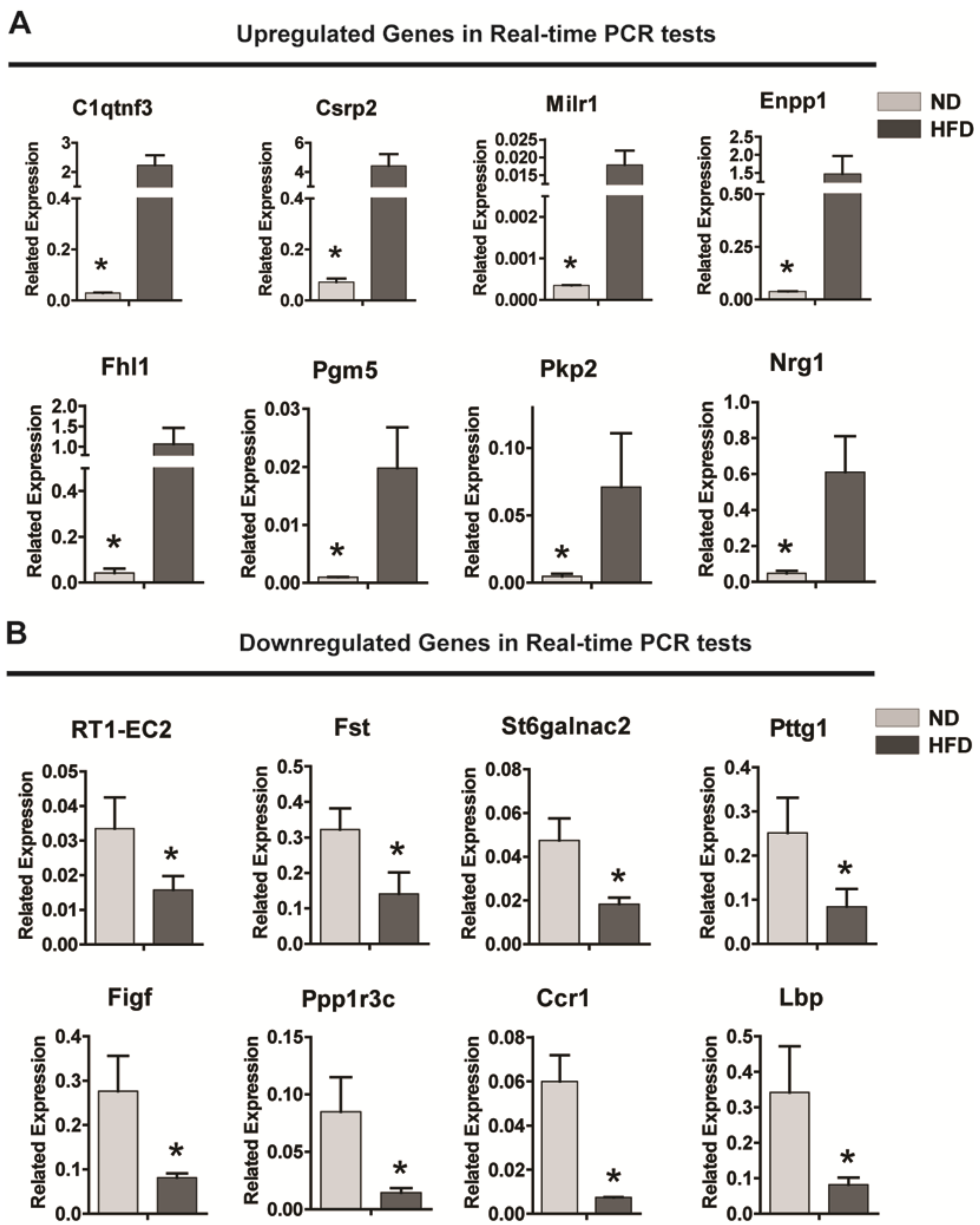

Supplementary Figure 2. Verification of microarray data with real-time PCR analysis. 16 representative differentially expressed genes (8 up- and 8 down-regulated) were verified with real-time PCR. Significance was calculated by student's t-test, $^{*}p < 0.05$.

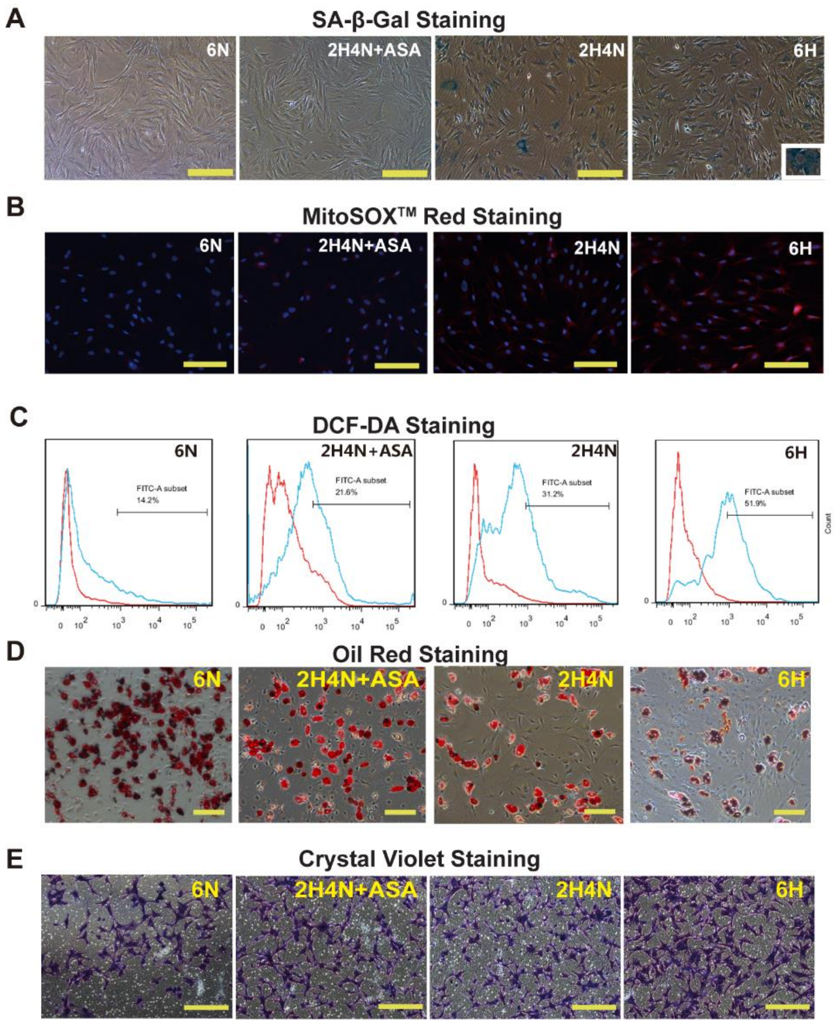

Supplementary Figure 3. Effects of aspirin and dietary intervention on BMSC functions. (A-E) SA-β-Gal staining (A), MitoSOX^{TM} Red staining (B), Flow cytometry analysis after DCF-DA staining (C), oil-red staining (D) and crystal violet staining (E) of BMSCs in the 6N, 6H, 2H4N and 2H4N+ASA groups. n=5. Scale bars, 50 μm.

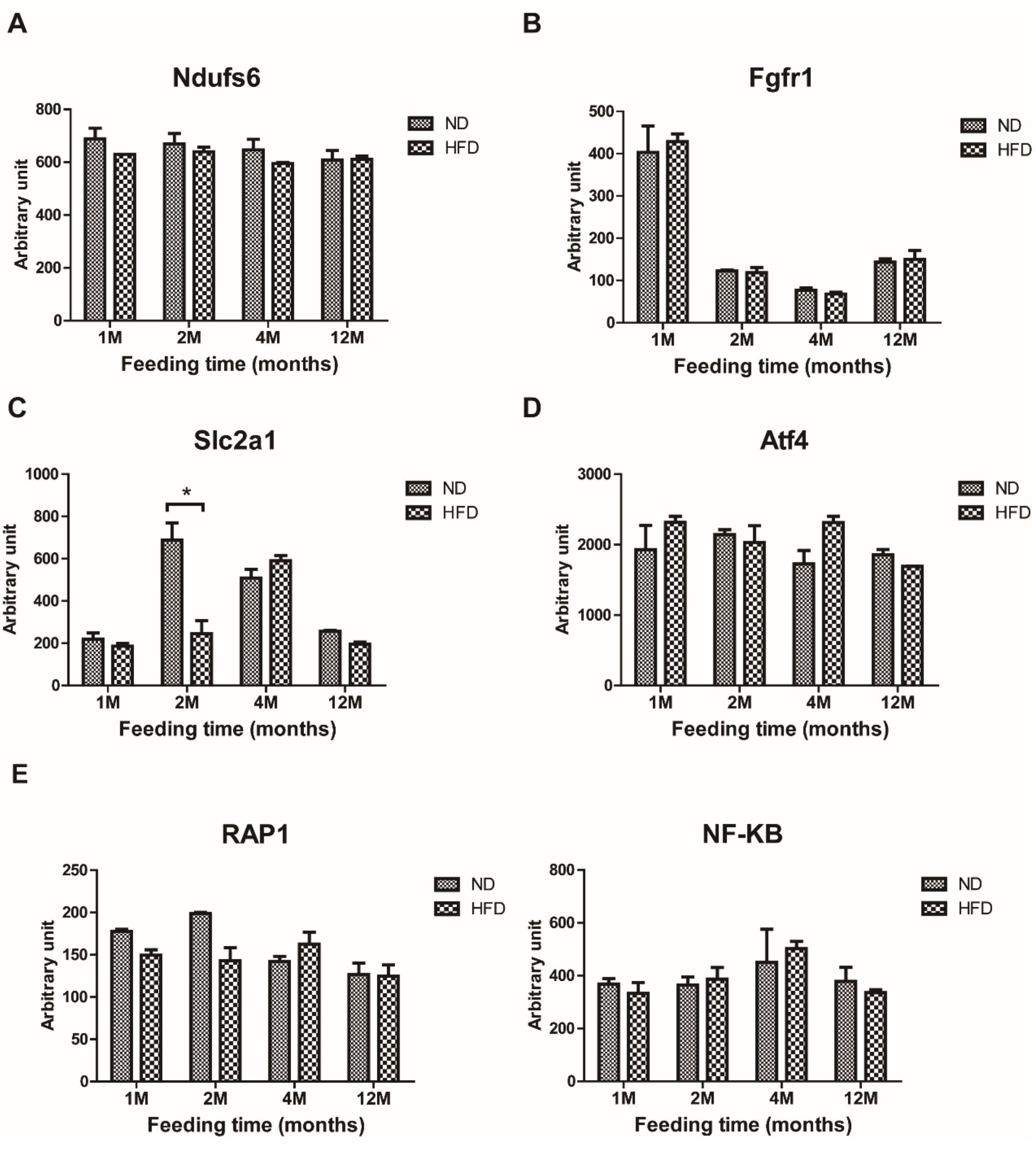

Supplementary Figure 4. Effects of HFD on the expression of genes involved in cell senescence and paracrine function of BMSCs. Microarray analysis was performed and the expression of senescence-related genes Ndufs6 (A) and Fgf21-signaling genes, including Fgfr1 (FGF21 receptor, B), Slc2a1 (FGF21 target, C) and Atf4 (transcription factor that positively regulate FGF21, D), and paracrine-related genes RAP1 and NF-κb (E) in BMSCs were summarized. Significance was calculated by student's t-test, $*p < 0.05$.
