# Supplementary material for: CXCL2 Impairs Functions of Bone Marrow Mesenchymal Stem Cells and Can Serve as a Serum Marker in High-Fat Diet-Fed Rats
Source: Front Cell Dev Biol. 2021 Jul 13;9:687942. doi: 10.3389/fcell.2021.687942 (PMC8315099; doi:10.3389/fcell.2021.687942)
Supplement: Supplementary file 2 [file Table_1.DOCX]

**Supplemenetary Table 1.** Nutrient Composition

Nutrient composition (per kg)

| Vitamin | | Mineral | | |
| --- | --- | --- | --- | --- |
| Vitamin A(IU) | 7800.00 | Na, (g) | 3.10 | |
| Vitamin D(IU) | 1200.00 | Mg, (g) | 2.90 | |
| Vitamin E(mg) | 67.00 | K, (g) | 7.40 | |
| Vitamin K(mg) | 5.00 | Cu, mg | 11.40 | |
| Vitamin B1(mg) | 10.00 | Fe, mg | 113.70 | |
| Vitamin B2(mg) | 15.00 | Mn, mg | 80.00 | |
| Vitamin B6(mg) | 10.00 | Zn, mg | 31.60 | |
| Vitamin B12(mg) | 0.02 | Se, mg | 0.20 | |
| nicotinic acid (mg) | 55.00 | Iodine, mg | 0.70 | |
| pantothenic acid (mg) | 22.00 | Energy composition | | |
| Biotin (mg) | 0.20 | Protein | 23.07% | |
| Choline (mg) | 1250.00 | Fat | 11.85% | |
| Folic acid (mg) | 6.60 | Carbohydrate | 65.08% | |
| Amino Acid | | Total energy | 3.40kcal/g | |
| Methionine+cystine (g) | 5.80 | Guaranteed nutritional value | | |
| Lysine (g) | 8.90 | Water | | ≤10% |
| Tryptophan (g) | 2.10 | Crude Fat | | ≥4% |
| Arginine (g) | 9.90 | Crude ash | | ≤8% |
| Leucine (g) | 14.80 | Crude Protein | | ≥18% |
| Isoleucine (g) | 7.40 | Crude Fiber | | ≤5% |
| Threonine (g) | 6.60 | Calcium | | 1.0-1.8% |
| Valine (g) | 8.90 | Phosphorus | | 0.6-1.2% |
| Histidine (g) | 4.90 |  | |  |
| Phenyl glycine+ tyrosine (g) | 14.60 |  | |  |
