# Supplementary material for: CXCL2 Impairs Functions of Bone Marrow Mesenchymal Stem Cells and Can Serve as a Serum Marker in High-Fat Diet-Fed Rats
Source: Front Cell Dev Biol. 2021 Jul 13;9:687942. doi: 10.3389/fcell.2021.687942 (PMC8315099; doi:10.3389/fcell.2021.687942)
Supplement: Supplementary file 3 [file Table_2.DOCX]

**Supplementary table 2**. Primer sequences used in real-time PCR analysis

| **Gene** | **Primer sequence 5'-3'** |
| --- | --- |
| *β-actin* | F:GAGAGGGAAATCGTGCGTGAC  R:CATCTGCTGGAAGGTGGACA |
| *Runx2* | F:TGAGATTTGTAGGCCGGAGC  R:AGGCGGTCAGAGAACAAACT |
| *OCN* | F:ATTGTGACGAGCTAGCGGAC  R:CCGTCCATACTTTCGAGGCA |
| *OPN* | F:CGGTGAAAGTGGCTGAGTTT  R:GGCTACAGCATCTGAGTGTTTG |
| *CEBP-α* | F:ATAAAGCCAAACAGCGCAAC  R:CGGTCATTGTCACTGGTCAA |
| *PPAR-γ* | F:CCCAATGGTTGCTGATTACA  R:GGACGCAGGCTCTACTTTGA |
| *C1qtnf3* | F:CCAGGCACTCCAGGGATAAA  R:GGCTACTTTCTGGGTAGCCT |
| *Csrp2* | F :ATGGAGGTGCTGAGAAGTGC  R :GCTCCTTGACCATAGCCGAA |
| *Milr1* | F :CCTCATGCAAGGGAGTGAGT  R :GACAGAGTAAGAGCCCAGAGTAAGA |
| *Enpp1* | F :GCCAGGATCAGACGTGGAAA  R :ACTGCTTACTGGTCCGTGTG |
| *Fhl1* | F :CTGGGCTTGAGAGAAGACGG  R :GCGCAGAACTTGTCAAAGCA |
| *Pgm5* | F :CAGAGTGTGCTGTCGTCCAT  R :GACTGCAGGTGTCGACAAGA |
| *Pkp2* | F :ACAATGTCACTGGATGCCTAAG  R :AATGAGACCATCACACCTCCTC |
| *Nrg1* | F :GATCAGCAAGTTAGGAAATGACAG  Reverse:CATGCCAGTGATGAACTCGT |
| *Uchl1* | F :ATTCAGGCAGCCCATGACT  R :AATGGAAATTCACTTTGTCGTCT |
| *Hisppd2a* | F :TGGTGGCTATCGGCTGTTTT  R :GGAGCTTTTTGCCATGGGTG |
| *Ankh* | F :GTGCCATTGCTGCAGTCTTC  R :ATTGAGGCACATCCCACCAG |
| *Hrasls* | F :CTGCCTTCTGGGGCACTAAG  R :GTGGGGGTTGTGAGGATAGG |
| *Fam101b* | F :CGAAGGAGTCGAGTTTGACC  R :AGAGTCGTACTTGACTGAGGAGGTA |
| *Gadd45b* | F :ATCCAATCGTTCTGCTGCGA  R :GCCTCGTTTGTGCCTAGAGT |
| *RT1-EC2* | F :CGTCTGTCACTCAGTCCACTC  R :CAAACATGAGGGGTGGGGAA |
| *Fst* | F :GCCTATGAGGGAAAGTGTATCAA  R :GCCAACCTTGAAATCCCATA |
| *St6galnac2* | F :ATCGATGCGCACGACTATGT  R :GGGGATGAAGATGTAGCGCA |
| *Sfrp1* | F :GCGCCCTCTTAATAAGCACA  R :GCACATGCGCAGACAGAC |
| *Figf* | F :TGTTTTACAAGATGAGAATCCACTG  R :GGGTTCCTGGAGGTAAGAGTG |
| *Lbp* | F :GTTACCGCCTGACTCCAACA  R :AGCTCCAAGTTCATGTCGGG |
| *Ppp1r3c* | F :GATGTTTGAAGTGGGGACTGA  R :ATGTGCAAGGTCCTGGATTC |
| *Ccr1* | F :AGGTTGGGACCTTGAACCTTG  R :TTCTGTGGTTGTGGGGTAGG |
